# Effect of Climatic Factors on Hand, Foot, and Mouth Disease in South Korea, 2010-2013

**DOI:** 10.1371/journal.pone.0157500

**Published:** 2016-06-10

**Authors:** Bryan Inho Kim, Hyunok Ki, Sunhee Park, Eunhi Cho, Byung Chul Chun

**Affiliations:** 1 Division of Infectious Disease Surveillance, Korea Centers for Disease Control and Prevention, Chungcheongbuk-do, Republic of Korea; 2 Department of Epidemiology and Medical Informatics, School of Public Health, Korea University, Seoul, Republic of Korea; University of Minnesota, UNITED STATES

## Abstract

Hand, foot, and mouth disease (HFMD) causes characteristic blisters and sores mainly in infants and children, and has been monitored in South Korea through sentinel surveillance since 2009. We described the patterns of HFMD occurrence and analyzed the effect of climatic factors on national HFMD incidence. Weekly clinically diagnosed HFMD case rates (per 1,000 outpatients) in sentinel sites and weekly climatic factors, such as average temperature, relative humidity, duration of sunshine, precipitation, and wind speed from 2010 to 2013, were used in this study. A generalized additive model with smoothing splines and climatic variables with time lags of up to 2 weeks were considered in the modeling process. To account for long-term trends and seasonality, we controlled for each year and their corresponding weeks. The autocorrelation issue was also adjusted by using autocorrelation variables. At an average temperature below 18°C, the HFMD rate increased by 10.3% for every 1°C rise in average temperature (95% confidence interval (CI): 8.4, 12.3%). We also saw a 6.6% increase in HFMD rate (95% CI: 3.6, 9.7%) with every 1% increase in relative humidity under 65%, with a 1.5% decrease in HFMD rate observed (95% CI: 0.4, 2.7%) with each 1% humidity increase above 65%. Modeling results have shown that average temperature and relative humidity are related to HFMD rate. Additional research on the environmental risk factors of HFMD transmission is required to understand the underlying mechanism between climatic factors and HFMD incidence.

## Introduction

Hand, foot, and mouth disease (HFMD) is caused by members of the genus *Enterovirus*, mainly by enterovirus 71 (EV 71) and different subtypes of Coxsackie virus, including CA16 [1]. HFMD is usually transmitted through direct contact with infected individuals and environmental factors [2]. The symptoms of this viral disease include blisters and sores in the mouth, on the palms of the hands, and on the soles of the feet, with a 3–7 day incubation period [3]. Although it is known to be a self-limiting condition from which a person can recover naturally, rarely, patients demonstrate severe complications, such as encephalitis, meningitis, and flaccid paralysis, which can lead to death, particularly in infants infected with EV 71 [4]. Although an EV71 vaccine has been developed [5] and was recently introduced in China [6], the vaccine’s availability is limited to China, and only symptomatic treatment is available in other countries. Thus, preventative measures have been emphasized to minimize the outbreak and transmission of HFMD.

HFMD has been a concern in Asian regions since the late 1990s [7,8], and it has been a major public health issue for neighboring countries including China and Japan [9–11]. Although EV71 was isolated in 2000 in South Korea [12], South Korea’s surveillance system for this disease was actually initiated in 2009 after several fatal cases were reported. Many previous studies have revealed that HFMD occurrence is associated with climatic factors in temperate countries [9] and that it has clear seasonality [9,13,14]. Cyclical patterns of 3 years have been observed in some countries [15]. Meteorological factors including temperature, relative humidity, and rainfall have been associated with HFMD in previous studies [16,17]. One study in China also revealed that HFMD was positively correlated with wind speed with a 1-week time lag [18]. Considering climactic factors in estimating HFMD incidence is critical in countries where HFMD is a major public health issue. In this study, we aim to analyze the pattern of HFMD occurrence and the effect of climatic factors on HFMD in South Korea. This is the first study using the HFMD sentinel surveillance data from the Korea Centers for Disease Control and Prevention (KCDC) to examine the associations between climatic factors and the incidence of HFMD in South Korea.

## Materials and Methods

### Study Area

South Korea is located in East Asia with a land size of 97,477 km^2^. According to Statistics Korea, in 2013 the total population of the country was approximately 51 million with a population density of 511 per km^2^. South Korea is part of the East Asian Monsoon region and has four distinct seasons with a temperate climate.

### HFMD Sentinel Surveillance Data

In 2008, 186 pediatric clinics voluntarily participated in the initial surveillance of HFMD. In June 2009, HFMD was designated as a national infectious disease that requires reporting by sentinel sites through a sentinel surveillance system. In the beginning of 2010, 193 sentinel sites were designated based on the population of each administrative region, and the number of participating clinics doubled to 393 in 2011 as the Infectious Disease Prevention and Control Act intensified HFMD surveillance. Sentinel sites were selected based on population size (1 site per 100,000 people). The number of sentinel sites was subsequently reduced to 100 in October 2013 (week 40). We did not include 2008 and 2009 surveillance data in the analysis, since reporting bias, such as underreporting was very likely during this introductory stage. Surveillance data after week 39 of 2013 was also excluded to minimize the effect of altered reporting facilities. Therefore, the study period consisted of 196 weeks (week 1 of 2010 to week 39 of 2013). Although the number of sentinel sites varied over the study period, these were selected based on the population of each region. HFMD was primarily diagnosed using the clinical characteristics of the disease such as mouth ulcers and vesicular lesions on particular regions that included the hand, foot, and buttocks. Surveillance data were directly reported from sentinel sites to the KCDC Division of Infectious Disease Surveillance through the web reporting system on a weekly basis. Hospitals participating in HFMD sentinel surveillance were only required to report the number of clinically confirmed HFMD patients out of the total number of outpatients once per week; therefore, the reports and data contain no personal information regarding the patients.

### Climatic Data

Average temperature, relative humidity, duration of sunshine, precipitation, and wind speed were analyzed from 16 climate centers from 2010 to 2013. These climate centers are located in 16 main cities and provinces of the nation, covering various geographical locations of South Korea. Daily climatic data were converted into weekly data to match the format of the surveillance data. Since the number of HFMD sentinel sites were based on the population of 16 regions, each climatic factor was also weighted using the number of HFMD sentinel sites. The climatic data were provided by the Korea Meteorological Administration.

### Statistical Analysis

Surveillance data consist of the number of clinically diagnosed HFMD cases and the number of total outpatients in each week. The total outpatient value is the number of outpatients having visited one of the primary pediatric sentinel sites to seek medical care for any reasons within the reporting week. The rate of HFMD is defined as the number of clinically diagnosed HFMD cases per 1,000 outpatients for the corresponding week. Pearson correlation analysis was initially attempted to identify an underlying correlation among climactic factors. Findings from the Pearson correlation analysis were taken into account in the subsequent modeling process. As there was no information about any underlying relationships, a generalized additive model (GAM) was applied. GAM with Poisson distribution was used to find the associations between the HFMD rates and climatic factors including average temperature, relative humidity, precipitation, duration of sunshine, and wind speed, all on a weekly basis. To account for seasonal and yearly variation, variables for individual weeks (1 to 196), weeks within the year (1 to 52 or 53), and individual years (2010 to 2013) were included in the model. The incubation period of HFMD (3–7 days), climatic data with a time lag of 1 or 2 weeks, and climatic data with a cumulative time lag (0–2 weeks) were also considered in the model. A lag between the time of HFMD onset and the actual reporting of the case through the sentinel surveillance system is likely; therefore, a time lag up to 2 weeks was taken into account in the modeling. Since HFMD rate was likely to have an autocorrelation issue by correlating with the preceding week, the variable representing HFMD rate with a 1-week time lag was incorporated in all models. To capture non-linear relationships, a smoothing spline with four degrees-of-freedom was used. When applicable, piecewise linear Poisson regression was used to determine the association between climactic factors and HFMD rate in detail. All statistical analyses were conducted using SAS 9.4.

## Results

### Descriptive Statistics

**Fig 1:**
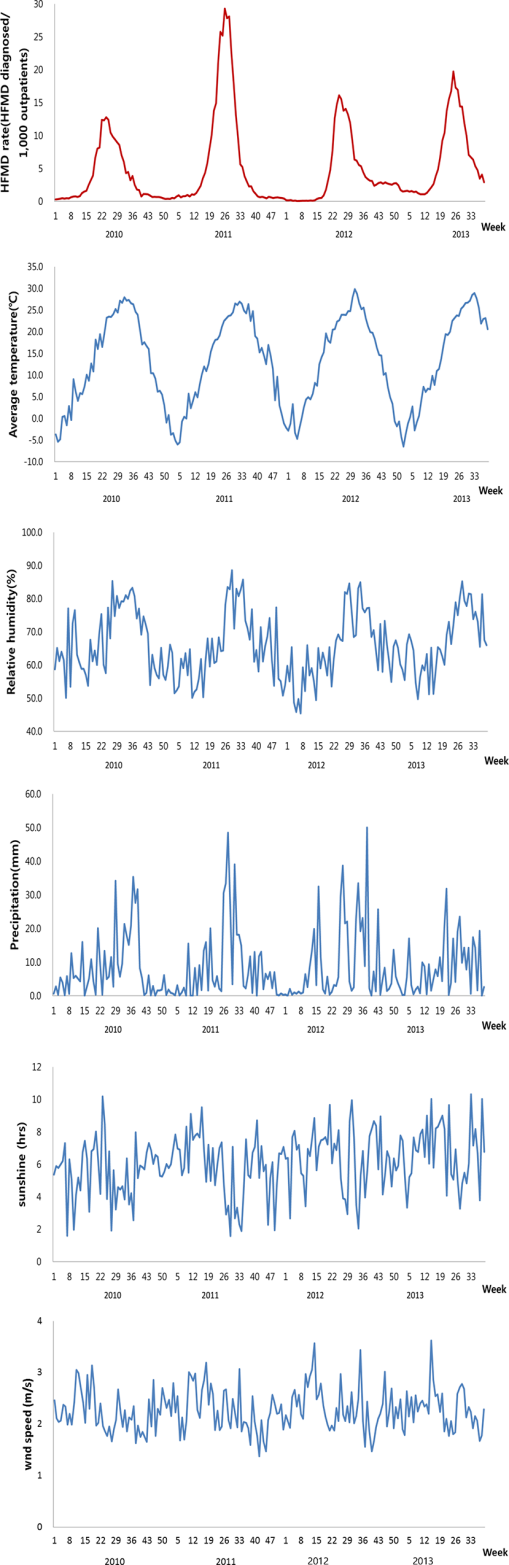
Weekly HFMD rate, average temperature, relative humidity, precipitation, sunshine, and wind speed in South Korea, 2010–2013.

During 40,461,309 outpatient visits at sentinel sites, 214,642 patients were clinically diagnosed with HFMD during the study period (0.53% of total outpatients). The weekly rate of HFMD ranged from 0.1 to 29.3, with a mean value of 5.0 (Table 1). Fig 1 shows the weekly climatic factors and HFMD rate in South Korea. The HFMD rate showed distinct seasonality over the study period; the disease was mainly reported from spring to early summer, peaking each year in late June.

**Table 1:** Descriptive statistics of the weekly HFMD rate and climatic factors, 2010–2013.

Variables	Mean	SD	Median	Min	Max
HFMD rate*	5.0	6.2	2.4	0.1	29.3
Average temperature (°C)	13.2	10.4	14.5	−6.5	29.8
Relative humidity (%)	66.0	9.9	65.2	45.4	88.5
Precipitation (mm)	8.5	10.0	4.7	0.0	50.0
Sunshine (hours)	6.1	1.9	6.2	1.6	10.3
Wind speed (m/s)	2.3	0.4	2.2	1.4	3.6

* Weekly HFMD cases per 1,000 outpatients

Simple correlation analysis revealed that the HFMD rate was positively correlated with average temperature, relative humidity, and precipitation, and negatively correlated with sunshine (Table 2). Among the climatic factors, relative humidity was highly correlated with other climatic variables, and it was not included in the same model with other factors, with the exception of wind speed, to avoid potential collinearity.

**Table 2:** Pearson correlation coefficient between HFMD rates and climatic factors, 2010–2013.

Climatic factors	HFMD rate*	Average temperature (°C)	Relative humidity (%)	Precipitation (mm)	Sunshine (hours)
Average temperature (°C)	0.61	-			
Relative humidity (%)	0.49	0.72	-		
Precipitation (mm)	0.39	0.47	0.62	-	
Sunshine (hours)	−0.15	NS	−0.63	−0.44	-
Wind speed (m/s)	NS	−0.22	−0.26	NS	NS

NS: Not statistically significant

* Weekly HFMD cases per 1,000 outpatients

### Regression Modeling Results

**Fig 2:**
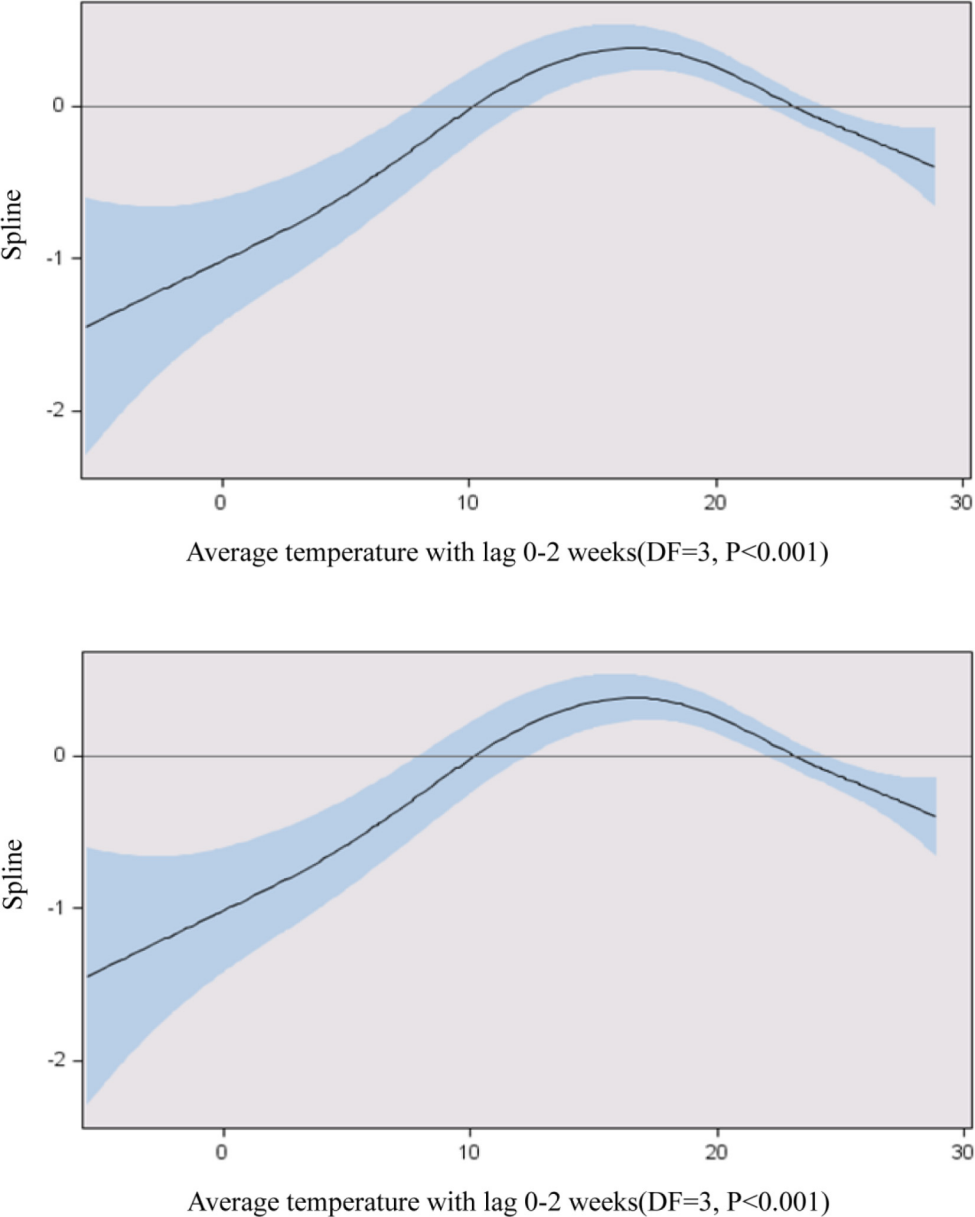
The effect of a 0–2-week time lag, average temperature, and relative humidity on HFMD rate controlling for seasonal and yearly variations. The line represents a spline curve and the shaded area shows the 95% confidence interval.

Time lags of up to 2 weeks were used in the GAM modeling, and the associations between each time lag climate variable and HFMD rate were analyzed. Only average temperature and relative humidity had any kind of pattern with HFMD rate in our analysis (Fig 2). Precipitation, sunshine, and wind speed had no significant effect on HFMD rate after controlling for long-term trends, yearly variation, and the autoregressive effect for HFMD rate; these variables were therefore dropped from the final model. The rate of HFMD was significantly associated with average temperature; specifically, increases in average temperature below 18°C had a positive impact on HFMD rate, while increases in average temperature above 18°C negatively influenced HFMD rate. This pattern was observed when analyzing average temperature with the other time lag variables (1 or 2 weeks and cumulative). Humidity, which is highly correlated with average temperature, was positively associated with HFMD rate when it was below 65%.

Based on these modeling results, a piecewise linear Poisson regression model was adapted with breakpoints at a temperature of 18°C and humidity at 65% using cumulative time lag climatic data (0–2 weeks). At an average temperature below 18°C, the HFMD rate increased by 10.3% (95% confidence interval (CI): 8.4, 12.3) for every 1°C rise in average temperature (Table 3). In addition to this, HFMD rate increased 6.6% (95% CI: 3.6, 9.7) per every 1% increase in relative humidity under 65%. Any additional 1% increase in relative humidity above 65% caused a 1.5% decrease in HFMD (95% CI: 0.4, 2.7).

**Table 3:** The effect of a 1-unit increase in average temperature and relative humidity on HFMD rate, with a cumulative time lag (0–2 weeks).

Climactic factors	Relative Risk	95% CI		P value
Average temperature <18°C	1.103	1.084	1.123	<0.0001
Average temperature ≥18°8	0.986	0.964	1.008	0.2146
Relative humidity <65%	1.066	1.036	1.097	<0.0001
Relative humidity ≥65%	0.984	0.973	0.996	0.0079

## Discussion

Although many studies have discovered associations between climatic factors and HFMD incidence in East Asia [8,19], this study is the first to explain HFMD occurrence in South Korea using nationwide HFMD sentinel surveillance data. When looking for associations, the long-term effects of seasonality and the short-term effects of climatic factors were taken into account. Unlike analyses from other countries that modeled average temperature and relative humidity together [13], this study used separate models for each climatic factor, since average temperature and relative humidity were highly correlated. South Korea is known to be very humid in the summer and dry in the winter. These climatic characteristics were identified in our data and were accounted for in the modeling process. In terms of piecewise regression analysis, average temperature and relative humidity were similarly associated with HFMD, probably due to the high correlation between these two climatic factors.

Modeling results have shown that average temperature and relative humidity are related to the HFMD rate in Korea. Specifically, HFMD rate rises along with average temperature and relative humidity up to certain points (18°C and 65%, respectively), and then subsequently decreases with increasing temperature and humidity. These findings are not consistent with other studies, which have shown either gradually increased HFMD incidence as the temperature rises [20] or a certain temperature range with an increased risk of HFMD transmission [21]. These discrepancies might be explained through human behaviors; it is extremely difficult to measure human behavior and quantify those patterns directly linked to HFMD transmission. Nevertheless, spring, with an average temperature of 11.7°C and average relative humidity of 60.9%, is the season during which outdoor activities increase significantly, and these kinds of behavioral changes and dynamics may facilitate HFMD transmission. It stands to reason that as temperature and humidity increase beyond 18°C and 65%, respectively, people begin to feel uncomfortable being outside and reduce their outdoor activities, accounting for the decrease in HFMD transmission in those conditions.

Although the effect of weather conditions on physical activity has been revealed in many studies [22], there have been no supporting studies about the effect of climatic factors on physical activity among Korean children. These explanations are based on assumptions, yet it remains the most likely situation given the limited data available. Population behaviors are likely to be different from other countries and could have affected the study results.

Potential confounders may have played major roles in the observed associations between climatic factors and HFMD incidence in South Korea. Further precise research is recommended to identify these possible confounders as well as the biological relationships between climactic factors and HFMD incidence in South Korea. With this regard, childcare centers are a potential confounder, since they are widespread and the cost is fully supported by the government. In these facilities, close contact with other children is likely to occur, which may contribute to the transmission of HFMD. Indeed, childcare centers were one of the risk factors for severe HFMD found in a neighboring country [23]. Further research is required to measure the effect of childcare centers on the spread of HFMD.

Unlike other studies that showed the effect of wind speed on HFMD incidence [13,18,24], wind speed was not associated with HFMD incidence in South Korea. Based on previous study results, it seems that each country and region has unique associations with climatic parameters, suggesting that countries with HFMD must determine their own relationships with various climatic factors.

The national sentinel surveillance system is operated based on designated nationwide sentinel sites. Although these sentinel sites were selected based on population size and are meant to represent the burden of HFMD in the nation, the data is limited in that the entire country’s occurrence of HFMD is not shown. Therefore, child population density may not be fully adjusted for, and this was one of the potential determinants for HFMD in China [17]. The number of sentinel sites also changed during the study period, and potential reporting bias is still likely, even though we used the number of outpatients as the denominator for determining HFMD rate. HFMD diagnosis is solely made upon clinical symptoms and no supporting laboratory test is available at this point. Although *Enterovirus* laboratory surveillance has operated since 2006, participants are mainly secondary and tertiary hospitals rather than primary clinics, and these hospitals are not population-based. This indicates that the existing laboratory surveillance system is not comparable to the HFMD sentinel surveillance system. Additionally, asymptomatic and mild HFMD cases can be missed, meaning that patients may not actively seek medical care. Thus, sentinel surveillance data may not fully estimate the total burden of HFMD in the nation. Still, the KCDC HFMD surveillance system is the only population-based nationwide sentinel surveillance system providing HFMD incidence data with population representativeness.

Predicting the effect of global warming on the occurrence of HFMD in Korea is not simple. If climate change on a global scale continues as it has been, the seasonality of HFMD is likely to be reduced, but overall occurrence is very likely to increase on a nationwide level. This would be comparable to other Southeast Asian countries in tropical regions where HFMD is regarded as an endemic disease that occurs throughout the year. This estimation is also supported by other studies that predict increased HFMD burden with climate change [19].

The history of HFMD surveillance in Korea is relatively short, and the nationwide primary clinic-based sentinel surveillance could be improved by appropriate supporting laboratory surveillance. This would enable the characterization of dominant *Enterovirus* strains and further genetic analysis of population-representative reported cases. In addition, spatial analysis in China has shown valuable information about HFMD [16,25]; therefore, this is one of the approaches that must be considered in future research. Although South Korea has a relatively small land size, spatial analysis can be used to identify the regions and populations most vulnerable to HFMD outbreak in South Korea. As HFMD is unlikely to be maintained under the current level in the near future, additional research into the environmental risk factors of HFMD transmission and additional efforts to incorporate various climatic factors in HFMD monitoring are required for more effective and efficient nationwide disease control.
